# Elite young soccer players have smaller inter-limb asymmetry and better body composition than non-elite players

**DOI:** 10.5114/biolsport.2023.114840

**Published:** 2022-04-21

**Authors:** Lucia Mala, Mikulas Hank, Petr Stastny, Frantisek Zahalka, Kevin R. Ford, Piotr Zmijewski, David Bujnovsky, Miroslav Petr, Tomas Maly

**Affiliations:** 1 Charles University in Prague, Faculty of Physical Education and Sport, Czech Republic; 2 Department of Physical Therapy, Congdon School of Health Sciences, High Point University, High Point, NC 27268, USA; 3 Józef Piłsudski University of Physical Education in Warsaw, Poland

**Keywords:** Fat mass, Fat-free mass, Physical performance, Soccer, Elite sport

## Abstract

Body composition (BC) and inter-limb anthropometric asymmetries (LA) may influence the physical performance of soccer players. This study aimed to determine differences in BC and LA among soccer across four performance levels. The study involved 110 male soccer players participating in Czech senior teams who were grouped into four different performance levels (i.e. G1: national team, G2: 1^st^ division, G3: 2^nd^ division, G4: 3^rd^ division). The following BC and LA parameters were compared among groups: body height, body mass, absolute fat-free mass, relative fat-free mass (FFMrel), percentage of fat mass (FM), total body water (TBW), intracellular water (ICW), extracellular water (ECW), phase angle, and bilateral muscle mass differences in the upper and lower extremities. Significant differences were observed in BC parameters among all groups (*λ* = 0.06, F75,246 = 5.38, p = 0.01, η_p_^2^ = 0.62). High-performance players (i.e. G1, G2) had significantly (p < 0.01) lower FM than lower performance players (i.e. G3, G4). The lowest values of FFMrel, relative TBW, relative ICW and ECW were detected in the lowest-performance players (i.e. G4). Significantly lower bilateral muscle mass differences were detected in G1 players (2.71 ± 1.26%; p < 0.01) compared with G4 players (3.95 ± 1.17%). G1 and G2 players had a higher proportion of muscle mass in the torso (p < 0.01) and upper limbs than G3 and G4 (p < 0.01). Elite and high-performance players have better BC and lower inter-limb anthropometric asymmetries compared with low-performance level players.

## INTRODUCTION

Elite soccer is a complex sport with a number of factors that may influence high performance, including technical and tactical skills, psychological factors, genetic predispositions [1] and physical fitness components [2] such as body composition (BC) [3]. Specifically, motor coordination and physical performance have been shown to discriminate between achievement level of soccer players [2, 4] within the meaning of current trends [5, 6, 7]. To achieve the highest sports performance and the opportunity to play in prestigious soccer competitions, players require long term preparation in specialized soccer programmes, mainly in talented youth development academies. About 16% of the academy players were promoted to the 1^st^ or 2^nd^ Spanish divisions in later years [6]. Although body size, muscle mass, maturation and physical performance (e.g. sprint, agility, endurance, jump test) have been found as limiting factors for career success in young soccer players [5, 8], no significant differences were found between players who remained members or left the academy. Conversely, no significant differences have been found within neuromuscular performance between successful and less successful players of the Spanish league [6] and elite and amateur players of the French league [9]. After a continuous selection process in the elite soccer academies, Los Arcos and Martins [10] reported that promotion from the reserve team to high-level soccer is not determined by the improvement in physical fitness performance in older players (age = 20.5 ± 1.5 years). On the other hand, higher 20-m dash sprint speed was evaluated between senior national team, junior national team, 1^st^ and 2^nd^ division players, and 3^rd^–5^th^ division, respectively [11]. Among above-mentioned physical characteristics, BC is an important mediator, which might provide desired differentiation between soccer performance levels.

Previous research reported that body fat mass (FM) is an important selection predictor for designated field positions (e.g. midfielders, defenders) and often distinguishes players who are highly or less successful [7, 12]. Elite players have a lower percentage of FM [13] and forwards are the leanest players among field positions [4]. On the other hand, no differences in FM were reported between professional and semi-professional Spanish players [14], particularly when comparing their specific field positions (e.g. defenders, lateral defenders, central defenders, midfielders, lateral midfielders, central midfielders, attackers). Some studies have indicated that higher-level soccer players have significantly different BC compared with the rest of the players [4], although this finding is inconsistent and non-significant in other studies [2, 12], probably due to the different approach of performance level separation. There have been studies that compared FM and fat-free mas (FFM) among different levels of soccer players [12, 4].

Generally, soccer players who are taller, heavier, more muscular, and have higher FFM with lower FM may have major advantages, especially during the growth and maturation period [15]. A significant negative correlation was reported between FM and covered distance during sprinting in official matches [16], which indicates that players with lower FM are able to cover longer distances in sprints and high-intensity running during the match. Absolute FFM strongly contributes to strength and power performance, and is considered a major prerequisite for good performance in professional soccer players [17]. However, the relationships between FM and other BC variables and their role in the success of players of various performance levels, ages, and genders in team sports, along with other lower limb strength performance factors, remain unclear [18].

The BC assessment aimed to distinguish and quantify different body segments and their properties [19]. In soccer, cyclic and acyclic movements are combined at irregular intervals, and athletes may show a higher-than-normal degree of morphological and strength asymmetry [20]. Asymmetries are an adaptive consequence that is increased by long-term and intense sports activities [21], and can thus indirectly indicate the risk of injury to a soccer player, in which a lower form of morphological asymmetry determines the occurrence of functional asymmetry. Many studies of soccer have been conducted on various aspects. However, most of the studies have been based on elite-level soccer, and there is limited research comparing four different performance levels (i.e. top, elite, sub-elite, amateur) with different BC variables, such as intra- and extracellular water and phase angle, as well as focusing on the detection of morphological asymmetries.

Since body BC studies do not consistently report differentiating parameters, distinguishing performance levels, which provide more consistent differences than physical fitness parameters, and BC symmetry relates to the injury prediction, this study aimed to determine differences in BC parameters and morphological limb asymmetry among four performance levels in youth soccer player. We hypothesized that there would be substantial differences in BC among different performance levels of young players, and higher morphological asymmetries in favour of low-performance level players.

## MATERIALS AND METHODS

### Subjects

The study involved 110 male soccer players (age, < 17 years), who were divided into four groups according to the level of performance as follows: G1_elite_, national team players (n = 20); G2_elite_, 1^st^ (highest) league players (n = 38); G3_sub-elite_, 2^nd^ league players (n = 32); and G4_sub-elite_, 3^rd^ division players (n = 20). The average period of soccer training experience for each group was 9.8 ± 3.6 years and typical training load during the week is shown in Table 1. The research was conducted from 2017 to 2020, during the pre-season player screening process. The basic anthropometric parameters of the players are included in the results (Table 2). The research design was approved by the Ethical Committee at Charles University, Faculty of Physical Education and Sport, and carried out according to the guidelines of the Declaration of Helsinki for human experimentation and the participants signed an informed consent form.

**TABLE 1:** Overview of a typical weekly training load (i.e. volume, frequency) for selected groups.

	G1_elite_	G2_elite_	G3 _sub-elite_	G4 _sub-elite_
	N = 20	n = 38	n = 32	n = 20
Field-Based Training	5 (60–80 min)	5 (60–80 min)	4–5 (60–90 min)	3 (80–100 min)
Resistance Training	2 (UB, LB)	2 (UB, LB)	1 (WB)	Core (as a part of field session)
Match	1–2 (90 min)	1–2 (90 min)	1 (90 min)	1 (90 min)

Legend: UB = upper-body resistance session; LB = lower body resistance session; WB = whole-body resistance session; G1_elite_ = national team players; G2_elite_ = 1^st^ division players; G3_sub-elite_ = 2^nd^ division players; G4_sub-elite_ = 3^rd^ division players.

### Data collection

Body height (BH) was measured using a digital stadiometer (Seca 242, Seca, Hamburg, Germany), and body mass (BM) was measured using a digital scale (Seca 769, Seca, Hamburg, Germany). Players were evaluated barefoot and wearing only undergarments. BC measurements were taken under the same conditions in the morning. In the 24 h prior to the measurement, the participants did not take any medications or pharmacological agents, including alcohol and caffeine, that may influence the results. They were also advised to abstain from food and fluids before the measurement, and to maintain good hydration and a normal routine in the interim. Furthermore, the athletes did not perform any high-intensity physical activity for a significant duration in the 48 h before testing. The room temperature was maintained between 20°C and 24°C to prevent undesirable changes in body water composition [22]. BC was assessed using a multi-frequency bioimpedance analyser (MC-980MA; Tanita Corporation, Tokyo, Japan), according to the manufacturer’s guidelines. Standardized conditions for bioimpedance measurements were maintained [23]. The BC indicators measured were as follows: BH, BM, FFM, relative fat-free mass (FFMrel), percentage of fat mass (FM), total body water (TBW), intracellular water (ICW), extracellular water (ECW), relative intracellular water (ICWrel), phase angle (PhA), muscle mass for the dominant and non-dominant lower limbs (MM_DL_ and MM_NL_), muscle mass for the dominant and non-dominant upper limbs (MM_DA_ and MM_NA_), and bilateral muscle mass (i.e. FFM) differences in the lower (ΔLE) and upper extremities (ΔUE). FFMrel was calculated as a normalized value of FFM to BM, and ΔLE, ΔUE were calculated as the percentage difference of the fat-free mass between the lower extremities.

### Statistical analysis

The differences in selected BC parameters among the observed groups were assessed using multivariate analysis of variance. We used Levene’s test for equality of variances to verify the homogeneity assumption that the variances of the dependent variable must be equal for all groups.

We used multiple comparisons of means (i.e. Bonferroni’s post-hoc test) to compare differences in specific parameters among the groups. When the criterion of sphericity was not met as an assumption for data processing, as assessed by Mauchly’s test (χ^2^), the degrees of freedom were adjusted using Greenhouse-Geisser sphericity correction, and statistical significance was evaluated based on the degrees of freedom.

The level of statistical significance was set at p < 0.05. The effect size was assessed using the partial eta square coefficient (η_p_^2^), which was the basis for the categorization of effect size as follows: small effect, η_p_^2^ = 0.02; medium effect, η_p_^2^ = 0.13; and large effect, η_p_^2^ = 0.26.

The p-value indicating the probability of a type I error (alpha) was set at 0.05, in all statistical analyses. Statistical analyses were performed using IBM SPSS v24 (IBM, Armonk, NY, USA).

## RESULTS

Multilevel analysis of variance revealed significant differences in BC parameters among all groups (*λ* = 0.06, F_75,246_ = 5.38, p = 0.01, η_p_^2^ = 0.62).

### Anthropometric parameters

The results showed (Table 2) no BH differences among the analysed groups (F_3,106_ = 0.67, p = 0.58, η_p_^2^ = 0.02), but a significant effect (F_3,106_ = 4.56, p = 0.01, η_p_^2^ = 0.59) of performance level on body mass, where the BM in G3_sub-elite_ was significantly lower than in G4_sub-elite_.

**TABLE 2:** Basic anthropometric parameters of the players (N = 110).

Variables		G1_elite_	G2_elite_	G3_sub-elite_	G4_sub-elite_	ANOVA			Post-hoc test
		n = 20	n = 38	n = 32	n = 20	F	p	η_p_^2^	
Body height (cm)	X (SD)	178.66 (6.63)	179.34 (5.67)	178.75 (5.70)	180.91 (5.96)	0.67	0.58	0.02	
Body mass (kg)	X (SD)	71.74(6.62)	70.83(7.56)	67.37(5.16)	74.46(8.38)	4.56	0.01	0.11	G3_sub-elite_ vs. G4 _sub-elite_

Legend: G1_elite_ = national team players; G2_elite_ = 1^st^ division players; G3_sub-elite_ = 2^nd^ division players; G4_sub-elite_ = 3^rd^ division players.

### Body composition

The FM percentage was significantly different among the groups (F_3,106_ = 50.10, p < 0.001, η_p_^2^ = 0.59), where G1_elite_ and G2_elite_ groups had significantly lower values than the G3_elite_ and G4 _elite_ groups (Table 3) and G3_elite_ had lower values than G4_elite_ (Table 3).

**TABLE 3:** Differences in body composition parameters among elite and sub-elite performance level groups.

Variables		G1_elite_	G2_elite_	G3_sub-elite_	G4_sub-elite_	ANOVA			Post-hoc test
		n = 20	n = 38	n = 32	n = 20	F	p	η_p_^2^	
FM (%)	X(SD)	9.92(1.44)	9.50(1.17)	13.47(2.41)	17.04(4.37)	50.09	< 0.01	0.59	G1 _elite_ vs. G3_sub-elite_, G4 _sub-elite_
									G2_elite_ vs. G3 _sub-elite_, G4 _sub-elite_, G3_sub-elite_ vs. G4 _sub-elite_

FFM (kg)	X(SD)	64.66(5.13)	64.88(5.79)	61.02(5.78)	61.42(5.58)	3.82	< 0.01	0.10	G2_elite_ vs. G3 _sub-elite_

FFMrel (%)	X(SD)	90.50(2.24)	91.58(4.37)	86.53(6.89)	83.00(4.70)	14.35	< 0.01	0.29	G4 _sub-elite_ vs. G1 _elite_, G2_elite_, G3 _sub-elite_

TBW (l)	X(SD)	45.59(4.87)	46.17(4.22)	43.95(3.41)	44.83(4.00)	1.82	0.15	0.05	

TBWrel (%)	X(SD)	63.54(3.10)	65.30(2.27)	65.33(3.56)	60.44(3.37)	13.57	< 0.01	0.28	G4 _sub-elite_ vs. G1 _elite_, G2_elite_, G3 _sub-elite_

ICW (l)	X(SD)	29.40(3.74)	30.04(3.26)	28.51(4.53)	26.90(2.40)	3.51	0.02	0.09	G2_elite_ vs. G4_sub-elite_

ICWrel (%)	X(SD)	64.37(1.61)	65.00(2.38)	64.75(3.72)	60.00(3.24)	5.48	< 0.01	0.13	G4 _sub-elite_ vs. G1 _elite_, G2_elite_, G3 _sub-elite_

ECW (l)	X(SD)	16.19(1.28)	16.13(1.47)	15.87(1.10)	17.93(1.60)	10.66	< 0.01	0.23	G4 _sub-elite_ vs. G1 _elite_, G2_elite_, G3 _sub-elite_

PhA1 (°)	X(SD)	7.09(0.56)	6.88(0.51)	6.25(0.67)	6.96(0.72)	10.59	< 0.01	0.23	G3_sub-elite_ vs. G1 _elite_, G2 _elite_, G4_sub-elite_

PhA2 (°)	X(SD)	7.13(0.59)	6.80(0.48)	6.24(0.67)	6.72(0.68)	10.83	< 0.01	0.24	G3_sub-elite_ vs. G1 _elite_, G2 _elite_, G4_sub-elite_

PhA3 (°)	X(SD)	6.79(0.42)	6.59(0.48)	6.28(0.42)	6.61(0.43)	6.08	< 0.01	0.15	G3_sub-elite_ vs. G1 _elite_, G2_elite_

PhA4 (°)	X(SD)	6.77(0.42)	6.53(0.50)	6.22(0.40)	6.56(0.49)	6.80	< 0.01	0.16	G3sub-elite vs. G1 elite, G2elite

Legend: FM = absolute value of fat mass; FFM = absolute value of fat-free mass; FFMrel = relative value of fat-free mass; TBW = total body water; TBWrel = relative value of total body water; ICW = intracellular water; ICWrel = relative value of intracellular water; ECW = extracellular fluid; PhA1 = phase angle right leg; PhA2 = phase angle left leg; PhA3 = phase angle right hand; PhA4 = phase angle left hand; G1_elite_ = national team players; G2_elite_ = 1^st^ division players; G3_sub-elite_ = 2^nd^ division players; G4_sub-elite_ = 3^rd^ division players.

FFM was significantly (F_3,106_ = 3.82, p = 0.01, η_p_^2^ = 0.10) higher in G2_elite_ in comparison to G3_sub-elite_, and FFMrel in G4_sub-elite_ group was significantly (F_3,106_ = 14.35, p < 0.01, η_p_^2^ = 0.29) lower than in the rest of the groups (Table 3).

Total body water (TBW) was not significantly different among the monitored groups (F_3,106_ = 1.82, p = 0.15, η_p_^2^ = 0.05). However, after comparing the TBW values according to body weight, the G4_sub-elite_ group had significantly (F_3,106_ = 13.57, p = 0.01, η_p_^2^ = 0.28) lower TBW values relative to body weight (TBWrel, %) than all other groups (Table 3). The body water components of ICW were higher (ICW: F_3,106_ = 3.51, p = 0.02, η_p_^2^ = 0.09) in the G2_elite_ group in comparison to the G4_sub-elite_ group (Table 3), and the G4_sub-elite_ group had lower (F_3,106_ = 5.48, p < 0.01, η_p_^2^ = 0.13) ICWrel (relative to ICW) values in comparison to the other groups at a higher performance level (Table 3). The ECW in the lowest performance group G4_sub-elite_ was significantly higher (F_3,106_ = 10.64, p < 0.01, η_p_^2^ = 0.23) in comparison to other groups (Table 3).

The G3_sub-elite_ group had lower values of PhA1(F_3,106_ = 10.59, p < 0.01, η_p_^2^ = 0.23) and PhA2 (F_3,106_ = 10.83, p < 0.01, η_p_^2^ = 0.24) than G1_elite_, G2_elite_ and G4_sub-elite_ (Table 3); and lower values of PhA3 (F_3,106_ = 6.08, p < 0.01, η_p_^2^ = 0.15) and PhA4 (F_3,106_ = 6.80, p < 0.01, η_p_^2^ = 0.16) than G1_elite_ and G2_elite_ groups.

### Segmental body fluid distribution

Analysis of the muscle mass volumes in the lower and upper limbs showed higher values (p < 0.01) of MM_DL_ and MM_NL_ between G2_elite_ vs. G3_sub-elite_ groups (Table 4), and lower values (p = 0.02) of ΔLE in G1_elite_ in comparison to G3_sub-elite_ group (Table 4). The G1_elite_ and G2_elite_ groups showed higher values of MM_DA_ (p < 0.01) and MM_NA_ (p < 0.01) in comparison to G3_sub-elite_, G4_sub-elite_ groups with no differences in ΔUE (p = 0.43) (Table 4). The trunk mass was lower in G3_sub-elite_ group in comparison to G1_elite_, G2_elite_ groups (Table 4).

**TABLE 4:** Comparison of segmental body fluid distribution among the elite and sub-elite performance groups.

Variables		G1_elite_	G2_elite_	G3_sub-elite_	G4_sub-elite_	ANOVA			Post-hoc test
		n = 20	n = 38	n = 32	n = 20	F	p	η_p_^2^	
MM_DL_ (kg)	X(SD)	10.81(0.95)	11.00(0.79)	10.24(0.90)	10.47(0.99)	4.68	< 0.01	0.12	G2_elite_ vs. G3_sub-elite_

MM_NL_ (kg)	X(SD)	10.52(1.02)	10.59(0.87)	9.84(0.90)	10.09(1.00)	4.56	0.01	0.11	G2_elite_ vs. G3_sub-elite_

ΔLE (%)	X(SD)	2.71(1.26)	3.73(1.70)	3.951.17	3.76(1.50)	3.33	0.02	0.09	G1 _elite_ vs. G3_sub-elite_

MM_DA_ (kg)	X(SD)	3.54(0.59)	3.78(0.47)	2.98(0.47)	2.96(0.47)	20.85	< 0.01	0.37	G1 _elite_ vs. G3_sub-elite_, G4_sub-elite_
									G2_elite_ vs. G3_sub-elite_, G4_sub-elite_

MM_NA_ (kg)	X(SD)	3.54(0.57)	3.73(0.48)	2.99(0.46)	2.98(0.45)	18.38	< 0.01	0.34	G1 _elite_ vs. G3_sub-elite_, G4_sub-elite_
									G2_elite_ vs. G3_sub-elite_, G4_sub-elite_

ΔUE (%)	X(SD)	1.69(1.88)	2.02(1.94)	2.53(2.32)	2.49(1.95)	0.93	0.43	0.03	

Trunk (kg)	X(SD)	32.51(2.80)	32.56(3.07)	29.99(2.49)	31.87(2.77)	5.73	0.01	0.14	G3_sub-elite_ vs. G1 _elite_, G2_elite_

Legend: MM_DL_ = muscle mass of dominant lower limb; MM_NL_ = muscle mass of non-dominant lower limb; ΔLE = bilateral muscle mass difference of lower limbs; MM_DA_ = muscle mass of dominant upper limb; MM_NA_ = muscle mass of non-dominant upper limb; ΔUE = bilateral muscle mass difference of upper limbs; G1_elite_ = national team players; G2_elite_ = 1^st^ division players; G3_sub-elite_ = 2^nd^ division players; G4_sub-elite_ = 3^rd^ division players.

## DISCUSSION

The analysis of basic anthropometric parameters did not reveal any difference in BH among performance groups, which agrees with the previous suggestion that BH may not be an individual criterion for career achievement, but it can be crucial in the selection for individual field positions [24]. According to Spanish league data, successful midfielders were taller and heavier than non-selected players [4] (selected: BH = 174.86 ± 6.98 cm, BW = 67.41 ± 8.19 kg; non-selected: BH = 170.45 ± 7.67 cm, BW = 62.10 ± 10.93 kg). This is contradicted by the Arnason et al. [2] study. Adult Iceland elite division players were significantly taller than division I players, which might be related to the local population and study size conditions. We noted that our highest performance players (G1_elite_) reached higher BH and BW (BH_G1_ = 178.66 ± 6.63 cm, BM_G1_ = 71.74 ± 6.62 kg) than the same age category players of the Belgian national team (176.8 ± 5.9 cm and 67.9 ± 6.7 kg, respectively) [25]. As the BH was not associated with the level of performance, the distinction is probably caused by the specific selection of the national team and individual extreme values at the time of evaluation.

Our study found that higher BM might be a performance disrupting factor, especially in combination with higher FM, ECW, and TBWrel, which was found in lower performance groups G3_sub-elite_ and G4_sub-elite_ (Table 3). This is in accordance with knowledge that soccer players with lower fat mass and higher lean mass may perform better than their lower-level peers in high-intensity intermittent running, change-of-direction speed, vertical jump, and sprint performance [26]. Several studies have investigated and confirmed negative correlations between increased fat mass and optimal muscle shortening, calcium signalling, physical performance, strength generation, sprint velocity, and aerobic power [16, 27 28, 29, 30, 31]. Although maintaining and developing active muscle mass and physical performance can potentiate higher anaerobic power output, systematically controlling and maintaining a lower FM does not increase excess BM. As a result, the muscles have a higher performance potential with a lighter resistance of the total BW [33].

FM does not seem to be a strict differentiating factor between elite levels, since we recorded no significant difference between the G1_elite_ and G2_elite_ groups [15, 34]. However, a significant difference in FM was observed between elite and sub-elite groups and between the G3_sub-elite_ and lowest performance group G4_sub-elite_ (Table 3). Our results are consistent with Reilly et al. [7], who reported higher FM in the sub-elite (13.9 ± 3.8%) than in elite soccer players (11.3 ± 13.9%) (age, 16.4 years; n = 31). Similarly, Gil et al. [4] reported significantly lower FM in selected players (11.43 ± 1.67%) than in non-selected players (12.56 ± 2.47%) for the defender position. However, no significant differences were observed among the different field positions (e.g. forwards, midfielders, and goalkeepers). These results may be due to the greater number of training hours per year in elite players (G1_elite_, G2_elite_) than in non-elite players. Vandendriesche et al. [25] reported that the youth Belgian national team (U17) had slightly higher FM (12.3 ± 3.0%) than our G1_elite_ and G2_elite_ groups (9.92 ± 1.44%). In adult players, there was no significant difference in FM between elite division players (9.9 ± 0.5%) and division I players (11.2 ± 0.5%) [2]. In addition, Rebelo et al. [34] reported no significant BC differences among elite (i.e. first division) vs. non-elite (i.e. regional division) Portuguese players in the under 19 years age category. Lower, although not significant, fat percentage was reported in successful teams than in unsuccessful young Spanish players (age, 15.63 ± 1.82 years) [12]. Male elite- and high-level soccer players typically undergo five to seven training sessions per week with up to one to two games per week, whereas in low- to medium-level players reach half of these values (Table 1). Thus, elevated FM values, along with lower levels of muscle mass, may negatively affect physical performance in youth soccer players [35].

The values of muscle mass and total fat-free mass recorded in our study showed similar values in players with higher performance levels (i.e. G1_elite_ and G2_elite_) and similar values in players with lower performance levels (i.e. G3 and G4). Figueireda et al. [32] reported a significant and positive correlation between the percentage of lean body mass and power performance (e.g. in the vertical jump) in professional players. Gil et al. [4] compared FFM values in successful (i.e. selected) and unsuccessful (i.e. non-selected) Spanish players (N = 231) at one level of competition, and found that, on muscle mass evaluation, there were no significant differences (p > 0.05) between the groups.

Monitoring the hydration status and body fluid volumes (i.e. TBW, ECW, and ICW) can help identify athletes who are at a higher risk of injury due to dehydration. Additionally, it can serve as the basis for prescribing fluid intake [36]. When estimating the distribution of fluids in terms of active muscle mass for individual body segments, the lowest bilateral morphological asymmetry was demonstrated in G1_elite_ players (< 3%), who had lower asymmetry than the G3_sub-elite_ group (Table 4). G2_elite_ players had a significantly higher volume of MM_DL_ and MM_NL_ than G3_sub-elite_ players. In connection with the non-significant difference in the basic anthropometric parameters, the obtained data imply a higher proportion of muscle mass in the lower limbs. The study findings also showed a higher proportion of upper limb muscle mass in the players from the G1_elite_ and G2_elite_ groups than in the players from the G3_sub-elite_ and G4 _sub-elite_ groups. The proportion of torso muscle mass in the G1_elite_ and G2_elite_ groups was also higher than that in the G3_sub-elite_ group. This result points to the importance of optimizing muscle mass and strength as influential factors for player contacts and fights, successful ball coverage, heading duels, and overall physical demands in elite soccer.

The quality of BC in terms of predisposition to a higher-level performance was also confirmed by a significant difference in the directly measurable PhA parameters and the indirectly estimated ICW value, in which the lowest values were found in the G3 _sub-elite_ group and the highest values were in the G1_elite_ group. The PhA values proportionally indicate muscularity in all players regardless of performance level and show the lateral preference of the kick leg. This difference was similarly observed in the upper limbs. Anaerobic performance measures showed that soccer players (age, 15 ± 1.4 years) with higher PhA values were faster in 10 m and 30 m sprints, and repeated sprint ability tests, and had higher ICW/ECW and TBW [37]. Independent of FFM, PhA has been shown to be positively correlated with maximum power and negatively correlated with the fatigue index [38]. In male soccer players, elite level players had significantly higher values of PhA and FFM than lower performance levels [39]. Some data suggest that as long as the cell hydration remains uncompromised and the ICW/ECW profile is unaffected, the reduction of FFM will not destabilize PhA levels and performance [40]. As this directly measurable variable may effectively evaluate athletes’ hydration status, cellular health, and physical function, there are limited recent studies which have reported on standard values for soccer athletes in different performance levels as well as research on PhA values among various sports [39, 41].

Mala et al. [15] reported the absence of morphological asymmetries in youth players (12–16 years old). In contrast, significant asymmetry was demonstrated in the elite players of the senior category, particularly goalkeepers, which was suggested to be due to unilateral limb preference in kicking and passing at greater distances during a game [42]. This hypothesis is also supported by Silvestre et al. [43], who recorded significantly lower FFM values in the dominant limb than in the non-dominant limb when comparing older players with younger players (p < 0.05). Focusing on asymmetry as a result of targeted and long-term sports activities, in which more experienced players tend to show greater asymme-tries than less experienced players [44], muscle asymmetry and muscle strength can be attributed to unilateral preference in most one-sided soccer skills [45]. Morphological asymmetry is subsequently followed by functional asymmetry. Authors report that the occurrence of strength asymmetry and imbalance between flexors and extensors is associated with a higher risk of injury [20]. Players who have bilateral asymmetries greater than 15% are up to five times more prone to ischiocrural muscle injury than players who have lower asymmetry.

The effect of morphological asymmetry was also confirmed in the study by Mala et al. [15], in which the analysis between soccer players aged under 17 years and soccer players in senior categories revealed significant differences in the morphological symmetry of the lower limbs (p < 0.01). Emphasis was placed on increasing asymmetries with aging and on the need for early diagnosis and compensation for unilateral loading. Achieving an optimal BC and favourable anthropometric variables may be advantageous for young players not only in terms of gaining greater muscle strength and power, but also in developing more efficient movement [46].

The generalizability of these results is subject to certain limitations. For instance, the cross-sectional study design does not permit us to draw conclusions about the future success of the players, since players may improve later in consecutive study designs (e.g. longitudinal study). Moreover, we did not distinguish the subjects according to field positions, although anthropometric and morphological differences among various field positions have previously been reported by several studies [3]. Further investigations involving other age categories are necessary to predict and determine the level of future success of a soccer player. Another limitation is that we only controlled for chronological age. In future studies, it will be necessary to control for biological maturation, possibly using a bio-banding approach [47], and sexual maturation (i.e. Tanner stage).

## CONCLUSIONS

The BC parameters and morphological asymmetries are related to the different performance levels, where high-performance players have high muscle mass in BC and lower bilateral asymmetry than low-performance players. Therefore, the training practice should emphasize high-quality and regular evaluations of BC, body hydration, and morphological asymmetry to enable early performance decrease, pathologic diagnosis and compensation for unilateral loading. Strength and conditioning or nutrition practitioners may also use the results of the present study to establish and create a tailored programme with the aim of achieving the most suitable anthropometric and BC profile for players at different performance levels.
